# siRNA-Mediated *Timp1* Silencing Inhibited the Inflammatory Phenotype during Acute Lung Injury

**DOI:** 10.3390/ijms24021641

**Published:** 2023-01-13

**Authors:** Ivan V. Chernikov, Yaroslav Yu. Staroseletz, Irina S. Tatarnikova, Aleksandra V. Sen’kova, Innokenty A. Savin, Andrey V. Markov, Evgeniya B. Logashenko, Elena L. Chernolovskaya, Marina A. Zenkova, Valentin V. Vlassov

**Affiliations:** Institute of Chemical Biology and Fundamental Medicine, Siberian Branch of the Russian Academy of Sciences, Acad. Lavrentiev Ave. 8, 630090 Novosibirsk, Russia

**Keywords:** acute lung injury, inflammation, chemically modified siRNA, TIMP1, IL6, LPS, bioinformatics analysis

## Abstract

Acute lung injury is a complex cascade process that develops in response to various damaging factors, which can lead to acute respiratory distress syndrome. Within this study, based on bioinformatics reanalysis of available full-transcriptome data of acute lung injury induced in mice and humans by various factors, we selected a set of genes that could serve as good targets for suppressing inflammation in the lung tissue, evaluated their expression in the cells of different origins during LPS-induced inflammation, and chose the tissue inhibitor of metalloproteinase *Timp1* as a promising target for suppressing inflammation. We designed an effective chemically modified anti-TIMP1 siRNA and showed that *Timp1* silencing correlates with a decrease in the pro-inflammatory cytokine IL6 secretion in cultured macrophage cells and reduces the severity of LPS-induced acute lung injury in a mouse model.

## 1. Introduction

Acute lung injury (ALI) is one of the leading causes of poor prognosis and death in critically ill patients. ALI is defined as an acute condition caused by severe non-cardiogenic factors such as severe infection, shock, and burns that result in diffuse interstitial and parenchymal pulmonary edema and is accompanied by a systemic inflammatory response. The clinical manifestations of ALI are progressive hypoxemia or acute respiratory distress syndrome (ARDS), which can progress to fatal respiratory failure and death [1,2,3,4,5,6,7,8,9,10].

Three million patients suffer from ALI/ARDS annually throughout the world, with very high mortality ranging from 35% to 46% [4,11]. Surviving patients face dire consequences and long recovery times, they are usually affected by muscle weakness and neuropsychiatric problems, so that less than 50% can return to work 12 months after leaving intensive care [12].

The main risk factor in ALI is uncontrolled inflammation, in which endothelial activation plays a central role. Depending on the cause (pulmonary or extrapulmonary), the inflammatory process can begin with damage to the alveolar and vascular endothelium. These events lead to diffuse inflammation in the lungs and impaired gas exchange. Inflammation leads to alveolar and interstitial edema, decreased alveolar fluid clearance, impaired surfactant production and function, and pulmonary fibrosis. The process affects both the alveolar and epithelial/endothelial pulmonary capillaries, resulting in inflammation and the production of cytokines by neutrophils, macrophages, monocytes, and lymphocytes. The release of inflammatory mediators from damaged lung tissue triggers systemic inflammation in the body, which can lead to multiple organ failure and death [13,14,15,16,17,18,19].

Nowadays, despite a better understanding of the pathophysiology, ALI/ARDS is typically diagnosed based on clinical and radiological features; there are no reliable laboratory markers to assess the progression of ALI, or effective treatments. The proinflammatory cytokines TNF-α, IL1β, IL6, IL8, and IL18 can be used as diagnostic markers for predicting morbidity and mortality [20,21], but they lack specificity. Currently, the main standard treatment options include mechanical ventilation to protect the lungs, prone position, the introduction of muscle relaxants, and the use of corticosteroids, but mortality from this condition is still high [22,23,24]. All of the above aspects demonstrate the need to improve the diagnosis and treatment of ALI/ARDS. Clinical trials of other pharmacological agents based on pathophysiological rationale, such as β2-agonists, statins, and keratinocyte growth factor, have not been beneficial and have shown possible deterioration [4].

Alternative approaches in drug design and ALI/ARDS treatment are required. Gene-targeted strategies for ALI/ARDS cure primarily focus on alveolar fluid clearance (AFC), alveolar capillary barrier function (ACBF), and pulmonary inflammation [25,26]. Enhancing AFC and restoring ACBF implies gene delivery (e.g., Na^+^, K^+^-ATPase β1 subunit) [25], while targeting pulmonary inflammation can be carried out by both gene delivery and gene silencing. The first strategy is to promote anti-inflammatory effects and implies the induction or delivery of anti-inflammatory cytokines, anti-oxidant enzymes, and other protective proteins, including IL10 [27], IL12 [28], superoxide dismutases (SODs) [29], heme oxygenase-1 (HO-1) [30], prostaglandins (PGs) synthase [31,32]. The second strategy is to fight against the overwhelming inflammation in ALI/ARDS by suppressing pro-inflammatory molecules using siRNA. Gene-targeted strategies based on the silencing of selected genes via siRNA may serve as a viable alternative to traditional pharmacological approaches [33,34,35,36,37]. Unfortunately, ALI/ARDS is characterized by large-scale rearrangements in gene expression profiles affecting dozens or even hundreds of genes [38] and the identification of key genes determining the molecular mechanisms of ALI/ARDS whose upregulation or downregulation could at least alleviate the symptoms and at best cure the disease is a complex and urgent task.

Neutrophil chemo-attractant Keratinocyte derived-chemokine (KC) and macrophage inflammatory protein-2 (MIP-2) are good examples of proteins that have the potential for siRNA therapeutics validated in animal models [39]. Nuclear factor kappa-light-chain-enhancer of activated B cells (NF-κB) is one of the most promising targets that serves as a central inflammatory mediator and regulates the expression of a number of downstream proinflammatory cytokines such as IL1b, IL6, and TNF-α, and sepsis-induced proinflammatory genes (iNOS, COX-2) [25,40,41,42]. Although NF-κB inhibition has been proved to be effective in suppressing inflammation in several studies [41,42,43], there are issues about the safety of the total inhibition of NF-κB because NF-κB is critical for maintaining normal immune responses [40,44,45]. Therefore, the search for new genes that could serve as both effective and safe targets for suppressing inflammation is of particular interest.

Within this study, we selected a set of genes that could serve as good targets for suppressing inflammation in the lungs tissue based on bioinformatics reanalysis of available full-transcriptome data of ALI induced in mice and humans by various factors, evaluated their expression in the cells of different origins during LPS-induced inflammation and chose tissue inhibitor of metalloproteinase *Timp1* as the promising target for suppressing inflammation. We constructed an effective anti-TIMP1 siRNA and examined the effect of Timp1 silencing on the secretion of the pro-inflammatory cytokine IL6 in cultured macrophage cells and the severity of acute lung injury in a mouse model.

## 2. Results

### 2.1. Selection of Inflammation-Associated Hub Genes as Potential Targets

**Figure 1:**
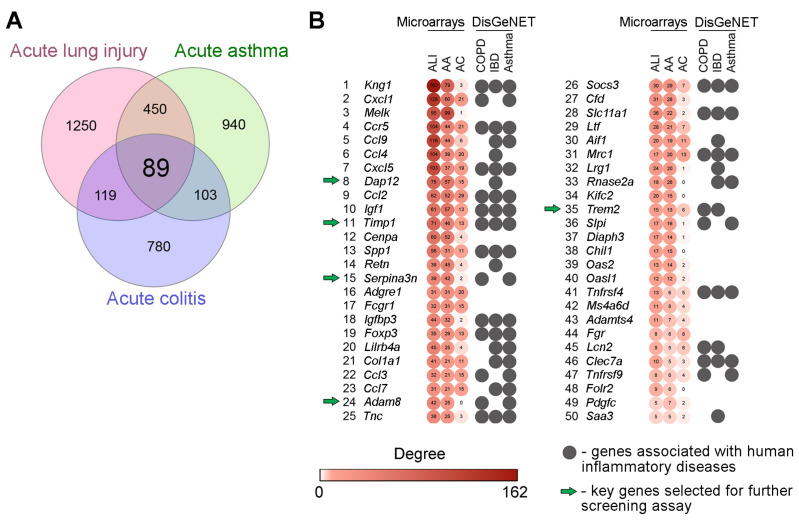
Core genes associated with acute inflammation: results of bioinformatics analysis. (**A**) Venn diagram illustrating the overlap between differentially expressed genes (DEGs) in inflamed lung and colon tissue of mice with ALI (GSE94522), acute asthma (GSE122197) and acute colitis (GSE35609). (**B**) Heatmap demonstrating the interconnection of revealed core genes in gene association networks reconstructed for each explored cDNA microarray datasets using the STRING database (confidence score ≥ 0.7) and their involvement in human inflammatory diseases according to DisGeNET database. Degree: the number of gene partners of DEGs within the gene network. Top-50 the most interconnected hub genes are presented. ALI—acute lung injury; AA—acute asthma; AC—acute colitis; COPD—chronic obstructive pulmonary disease; IBD—inflammatory bowel disease.

In the first step of the study, we questioned which genes are readily susceptible to pro-inflammatory stimuli of various origins and occupy the hub positions in inflammatory-associated regulome. To address this, two independent expression profiles of inflamed lung tissues from mice with bleomycin-stimulated ALI (GSE94522) and ovalbumin-induced asthma (GSE122197) were retrieved from the GEO database. Next, to exclude genes that probably execute organ-specific functions and, thus, to concentrate our effort on the search for key genes common to inflammatory pathological processes in general, expression profile of colon tissue from mice with dinitrobenzene sulfonic acid-driven colitis (GSE35609) was also involved in the current study. Analysis of listed transcriptomic datasets using GEO2R tool revealed the sets of differentially expressed genes (DEGs) (pathology vs. healthy control), further overlapping of which revealed 89 common DEGs specific for all explored inflammatory disorders and, thus, forming a list of promising core genes susceptible to the response to various phlogogenic stressors (Figure 1A).

Next, in order to evaluate how deeply revealed core genes are interconnected with inflammation-perturbed lung and colon transciptomes in mice, gene association networks were reconstructed from all computed ALI-, asthma- and colitis-related DEGs using STRING database followed by the analysis of their topology. Inflammation-associated core genes were ranked according to their degree centrality scores and the top-50 hub genes the most interconnected with reconstructed gene networks are depicted in Figure 1B. Obtained results clearly demonstrated that hub positions in inflammatory regulomes were mainly occupied by the genes encoding well-known modulators of the inflammatory response, including chemokines and their receptors (*Cxcl1*, *Ccl9*, *Ccl4*, *Cxcl5*, *Ccl2*, *Ccl3*, *Ccl7*, *Ccr5*), kininogens (*Kng1*), adipokines (*Retn*), and the components of the immunoglobulin response (*Fcgr1*, *Lilrb4a*) and insulin-like growth factor signaling (*Igf1*, *Igfbp3*), which showed the adequate reliability of our results obtained using an in silico approach. Further annotation of revealed list of core genes using the DisGeNET database demonstrated their involvement in the progression of a range of inflammatory disorders in humans, including chronic obstructive pulmonary disease (COPD) (*p* = 1.03 × 10^−9^), asthma (*p* = 6.61 × 10^−13^), and inflammatory bowel disease (IBD) (*p* = 4.03 × 10^−16^) (Figure 1B), which demonstrates, firstly, the presence of a translational bridge between explored inflammatory disorders in mice and humans and, secondly, expediency of further evaluation of identified core genes as promising targets for gene silencing therapy. Given the high degree centrality score, significant enrichment with inflammatory diseases in DisGeNET, and limited understanding of the role in the immune response, the following hub genes, were selected for further steps of the study: *Dap12*, *Timp1*, *Serpina3n*, *Adam8*, *Trem2.*

A membrane adaptor DNAX-activating protein of 12 kDa (Dap12, also known as Tyrobp and Karap) is expressed by a variety of innate immune cells, including dendritic cells, natural killer (NK) cells, microglia, monocytes, and macrophages [46], suggesting a general function in immune responses [47]. The role of Dap12 is quite ambiguous. Dap12 is involved in both infectious and non-infectious inflammation of different etiologies and can either inhibit or activate inflammation [48,49,50,51].

Tissue inhibitor of metalloproteinase *Timp1* is one of the most promising genes associated with inflammation [52,53,54,55,56]. Timp1 works as an inhibitor of matrix metalloproteinases including for example Mmp9, the major enzymes responsible for the remodeling of the extracellular matrix (ECM), a process that is significantly activated in conjunction with inflammation [57,58,59,60]. Besides the well-recognized Mmp inhibition functions of all four members of the Timp family include various cytokine-like activities acting as signaling molecules. In that capacity Timp1 can modulate cell proliferation, apoptosis, differentiation, and angiogenesis [53].

Serpins (serine peptidase inhibitors) are a superfamily of proteins involved in the control of blood coagulation, complement activation, programmed cell death via maintaining an appropriate level of peptidase activity an imbalance of which may result in various lung abnormalities including asthma [61], COPD [62], ARDS [63,64]. Serpina3 is one of the most abundant serpins (along with *Serpina1*, alpha(1)-antitrypsin, AAT), up-regulated by 4-5-fold during inflammation [65,66]. Although a major producer of Serpina3 is the liver, other organs including the lungs are also capable of synthesizing it [65].

A disintegrin and metalloproteinase 8 Adam8 is an enzyme that participates in the recruitment of leukocytes from the pulmonary blood vessels into the lung tissue during acute lung inflammation [67,68,69]. Adam8 is not regulated by Timp1 or other Timps [70,71] such as matrix metalloproteinases and thus may a represent relatively independent way of regulating of inflammation.

We also included in the study genes encoding the triggering receptor expressed on myeloid cells Trem2 (hub gene; 35 position) (Figure 1B) and metalloproteinase Mmp9, that are integral elements of well-known inflammatory-associated axes Dap12-Trem2 [47] and Timp1-Mmp9 [72]. Additionally, component C3, the cornerstone of a complement system and the multifunctional immune mediator placed in the crossroad of several pathways [73], which are involved in the pathogenesis of various inflammatory lung diseases, including asthma [74], COPD [75], infectious disease [76], and bleomycin-induced fibrosis [77] was also chosen for further study. Thus, the list of selected inflammation-related potential targets included *Dap12*, *Timp1*, *Serpina3n*, *Adam8*, *Trem2*, *Mmp9*, and *C3*.

### 2.2. Screening for the Expression of Selected Genes in Cell Lines of Different Origin Stimulated by LPS

**Figure 2:**
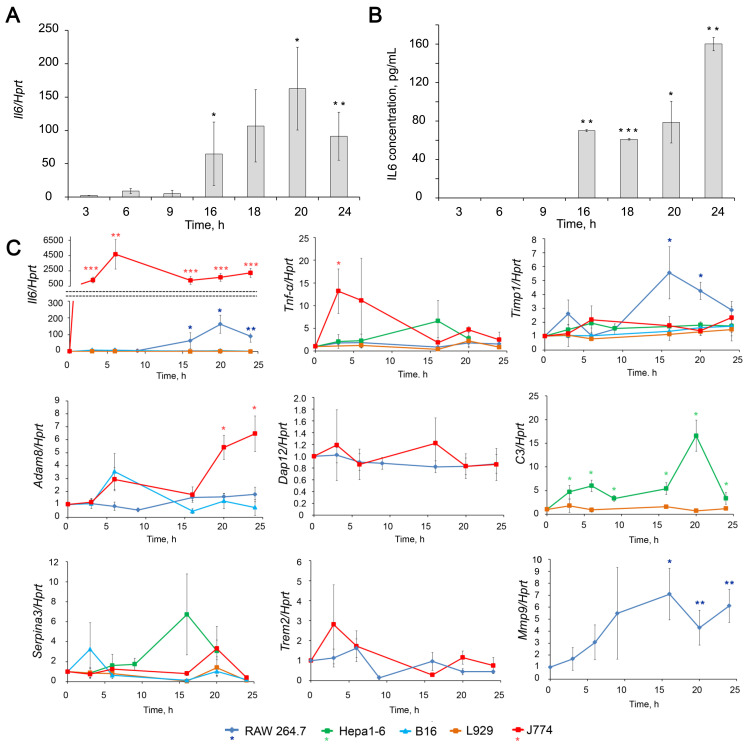
Dynamics of expression level changes of inflammation-associated genes in different cell lines upon LPS stimulation. (**A**,**B**) Levels of *Il6* mRNA (**A**) and secreted IL6 (**B**) in LPS (1 µg/mL) stimulated RAW264.7 cells. *Il6* mRNA level was measured by RT-qPCR, and its ratio to the level of hypoxanthine phosphoribosyltransferase (*Hprt*) mRNA was normalized to the ratio in control, non-treated cells. Il6 protein level was measured using ELISA. (**C**) Relative levels of *Il6*, *Tnf-α*, *Timp1*, *Adam8*, *Dap12*, *C3*, *Serpina3* and *Mmp9* mRNA in different cell lines stimulated by LPS measured by RT-qPCR normalized to the level of *Hprt* mRNA. The level in control, non-treated cells was set as 1. The exact fold changes, confidence intervals and statistical significance are given in Supplementary Materials Table S1. * *p* ≤ 0.05, ** *p* ≤ 0.01, *** *p* ≤ 0.001, Student’s two-tailed unpaired *t*-test.

Creating an efficient silencing tool, either siRNA or antisense oligonucleotides (ASO), requires screening a set of promising candidates using an appropriate cell model for each gene. Expression levels of selected genes (*Timp1*, *Adam8*, *Dap12*, *C3*, *Serpina3*, *Trem2*, *Mmp9*) and pro-inflammatory cytokines interleukin-6 (*Il6*) and tumor necrosis factor α (*Tnf-α*) were assessed in cell lines of different tissue origins (RAW264.7, J774, Hepa 1–6, B16, and L929) stimulated with lipopolysaccharide (LPS). LPS from Gram-negative bacteria has a wide spectrum of action, demonstrating a pro-inflammatory effect and can be used to induce inflammation both in vitro and in vivo [78,79]. Primarily, we tested cell lines that are directly related to inflammation (macrophages). Since some of the genes that were selected according to the bioinformatic analysis were not expressed at a detectable level in cells of macrophage origin, even after LPS stimulation, we added to the list the cells originating from organs and tissues for which inflammation is directly associated with the development of pathology (hepatoma, fibroblasts). Melanoma was taken for comparison because there was no data about its association with inflammation. Expression levels of *Il6* and *Tnf-α* served as a positive control, confirming cell activation in response to inflammatory stimuli. The measurements were carried out at 3, 6, 9, 16, 20, and 24 h time points to find an optimal time interval for silencing each gene (Figure 2, Supplementary Table S1).

Upon LPS stimulation, RAW264.7 macrophages produce the inflammatory cytokines IL6 and, to a lesser extent, TNF-α (Figure 2). We have shown that the kinetics of *Il6* activation includes two phases starting from moderate (2–14 folds, 3-6 h after LPS addition) activation, followed by a phase of intense activation (90–107 folds, 16–24 h after LPS addition). The mRNA level of *Il6* positively correlates with the protein level assessed by ELISA (Figure 2). Another mouse macrophage cell line, J774, showed a huge 1300–4700-fold increase in *Il6* mRNA expression levels without clear phase separation. This result complemented the previously obtained IL6 release data [80]. Thus, J774 cells showed an even more pronounced response to the inflammatory stimulus than RAW264.7. Hepa1-6 and B16 cells exhibited a less pronounced response to LPS induction; however, the expression level of *Il6* increases 2–10 times with a maximum observed at 3 h. As we expected, no activation of *Il6* mRNA expression was observed in L929 cells in response to LPS induction (Figure 2) since L929 cells were considered as cells insensible toward LPS stimulation.

Tumor necrosis factor α (TNF-α), which serves as a second indicator of inflammation, slightly increased upon LPS stimulation by in RAW264.7 macrophages (1.4 fold on average) and remained at the same level or very slightly increased in Hepa 1–6, B16 and L929 cells. J774 macrophages were the only cell line in which *Tnf-α* mRNA expression showed a noticeable 7–25-fold increase 3–6 h after LPS stimulation, followed by a decline to 1.2–5-fold within 6–24 h (Figure 2C).

Expression level of *Timp1* increased in RAW264.7 upon LPS stimulation showing bell-shaped kinetics *Timp1* mRNA level raised 1.5–3.5-fold during the first 3–9 h, then increased up to 4.3–9.7-fold at 16–20 h, followed by partial decline to 2.6–3.6-fold to 24 h. An increase (by 1.5–2 fold) of *Timp1* mRNA level in response to LPS stimulation was also found in J774, Hepa1-6, and B16 cells, although to a lesser extent compared to RAW264.7 cells (Figure 2C).

The *Mmp9* expression in RAW264.7 cells shows kinetic compatible with that of *Timp1:* continuously increasing from 3 to 16 h and reaching maximum (7 fold) at 16–24 h. TIMP1 is a natural inhibitor of MMP9, and the concurrent expression of these two genes is well known [72,81,82]. As for MMP9, we did not consider it a target since its activation is not a cause but a consequence of pathological processes and is necessary for the resolution of inflammation. This gene is of interest because of its functional relationship with TIMP1.

The mRNA expression levels of *Adam8* did not change significantly upon LPS stimulation in the cell line being studied except J774, where *Adam8* was found up-regulated up to 2–12-fold within 6–24 h. No significant changes were detected in the expression of *Dap12* in the cell lines under study.

Hepa1–6 appeared to be the only cell line out of studied, in which the mRNA level of *C3* (Figure 2C) was significantly 3–17-fold increased with a maximum at 20 h time point. In the other cell lines, expression of *C3* was not detected nor did not change after LPS stimulation.

An increase (4–8 fold) in *Serpina3* expression level was observed in Hepa1-6 cells at 16–20 h, and in J774 cells at 20 h (Figure 2C). *Trem2* appeared to be the only gene that showed a tendency to down-regulation in RAW264.7 cells upon LPS stimulation.

**Figure 3:**
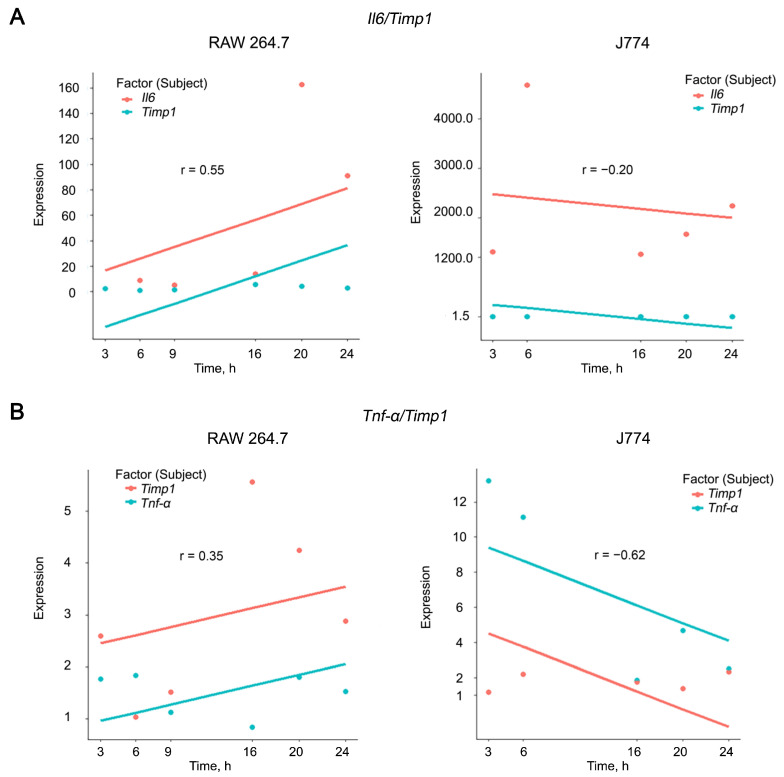
Correlation analysis of expression levels of *Il6* (**A**), *TNF-α* (**B**), and *Timp1* in RAW 264.7 and J774 cell lines after LPS stimulation. Correlation coefficients were calculated using rmcorr package in R. Plots were constructed using the ggplot2 package in R. *x* and *y*-axis breaks on the plots were introduced using the ggbreak package in R. Correlation coefficients were determined as follows: strong correlations 0.70 ≤ r ≤ 1.00, moderate correlations 0.30 ≤ r ≤ 0.69, weak correlations 0.01 ≤ r ≤ −0.29.

Thus, the most pronounced increase in the expression levels of the pro-inflammatory cytokines *Il6* and *Tnf-α* was observed in macrophages and was accompanied by a significant activation of the expression of the *Timp1* and *Mmp9* genes. To confirm this observation, we calculated correlations between the expression levels of IL6, TNF-α, and TIMP1 in RAW 264.7 and J774 cell lines using rmcorr package in R [83]. Correlation analysis was visualized using the ggplot2 and ggbreak packages [84]. It appeared that RAW 264.7 cells were characterized by weak (r = 0.35) to moderate (r = 0.54) positive correlations in the case of TNF-α/Timp1 and Il6/Timp1 combination, respectively, while J774 cell line displayed a reverse trend of weak (r = −0.2) and moderate (r = −0.62) negative correlations in the case of Il6/Timp1 and TNF-α/Timp1 combination, respectively (Figure 3 and Supplementary Figure S2).

Obtained data let us suggest that *Timp1* is the best candidate target among the genes under the study for suppressing inflammation. The presence of both positive and negative correlations with the expression of pro-inflammatory cytokines reflects a dual relationship with their production: on the one hand, *Timp1* has long been recognized to be regulated by *Il6* [81,85,86,87], but at the same time *Timp1* has been recently proved to be a regulator of *Il6* [88]. Based on our results, we can make a prudent assumption that Timp1 more likely regulates Il6 than vice versa.

### 2.3. Functional Analysis of Timp1 and Its First Neighbors in Gene Networks

**Figure 4:**
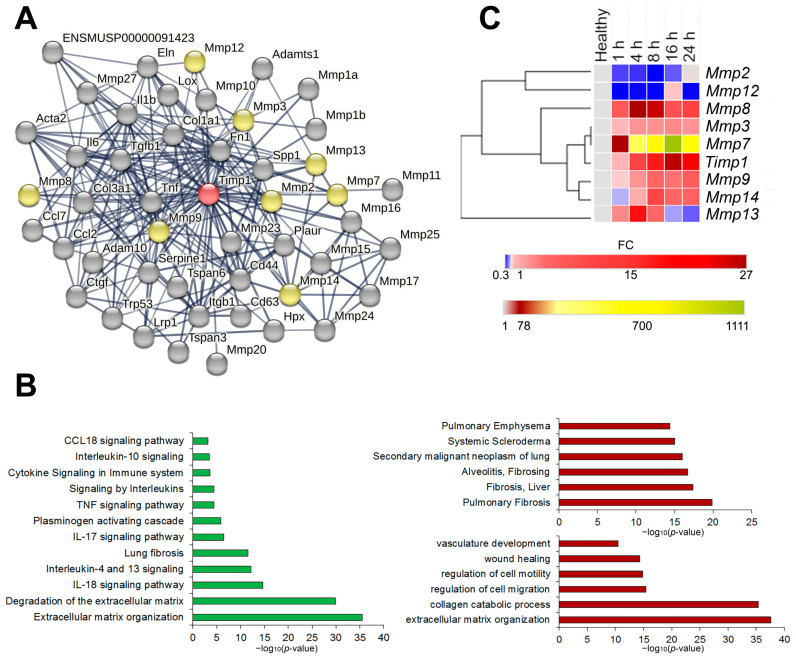
Identification, functional and expression analysis of Timp1 and its first neighbors. (**A**) Protein–proteins interaction network of Timp1 and its first neighbors reconstructed using the STRING database web-service. No more than 50 highest confidence first neighbors are shown; minimum confidence score of the interators > 0.700. The following interaction sources were used: text mining, experimental data, databases, co-expression, neighborhood, gene fusion and co-occurrence. Red colored node: query gene; yellow colored nodes: first shell interactors from MMP family; gray nodes: all other first shell interactors. (**B**) Histograms visualizing the functional enrichment analysis of Timp1 and its first neighbors. Functional enrichment analysis was performed through the ToppFun module of the ToppGene suite, using the MSigDB Biocarta (v.7.5.1), BioSystems: Reactome and KEGG, Panther DB (**left panel**), DisGeNET BeFree and DisGeNET curated (**right upper panel**) and Gene Ontology: biological processes (**right lower panel**) databases. For all enrichment tools, the input gene set was composed of the same 44 genes and default options were used: significance threshold of 0.05 for Bonferroni adjusted *p*-value, at least five genes from the input list in the enriched category, and the whole genome as background. (**C**) Dynamics of expression levels of *Timp1* and interconnected *Mmp*s in the lung tissue of mice with LPS-induced acute lung injury at different time points. Heat map construction was performed using Morpheus. FC = fold change.

In order to analyze the possible interconnections of *Timp1* to the inflammatory processes in the lungs, identification and functional analysis of *Timp1* and its first neighbors in gene networks with subsequent validation on the murine model of LPS-induced acute lung injury at different time points were performed (Figure 4).

We identified 47 first neighbors of Timp1 in the STRING database with high (>0.700) confidence scores using the following interaction sources: text mining, experimental data, databases, co-expression data, neighborhood, gene fusion and co-occurrence (Figure 4A). Functional annotation of the identified genes using ToppFun web-service (Figure 4B) have demonstrated close association of the identified genes with processes, linked with extracellular matrix (ECM) degradation, organization and subsequent development of pulmonary fibrosis, with both pro- (Il-10, Il-18, Ccl18, Tnf pathways) and anti-inflammatory (Il-4 and Il-13 pathways) signaling (Figure 4B, left panel) Analysis of databases DisGeNET Curated and DisGeNET BeFree has shown that the identified genes are involved in the development of chronic inflammatory diseases of lungs and liver, as well as malignant neoplasms of the lungs (Figure 4B, right upper panel). Finally, the analysis of the Gene Ontology: biological functions database indicates involvement of the aforementioned genes in the vasculature development, ECM organization and regulation of cell motility/migration (Figure 4B, right lower panel). Thus, we observe the close involvement of the *Timp1* gene and its first neighbors both in acute inflammation and its long-term consequences such as fibrosis development and tumor transformation.

Validation of bioinformatics analysis findings was performed on the murine model of LPS-induced ALI at different time points during the first 24 h after induction by RT-qPCR. The expression level profiles were analyzed by hierarchical clustering using the one minus spearman correlation rank method (Figure 4C and Supplementary Figure S1). *Timp1* and several *Mmp* genes, functionally directly related to *Timp1*, were selected for validation. Analysis of gene expression patterns revealed that *Timp1* expression increases steadily during the first hours, reaching its peak 16 h after induction. Of all analyzed *Mmp*s, *Mmp3* and *Mmp7* expression profiles were the closest to that of *Timp1* (Figure 4C). *Mmp8*, *Mmp9*, and *Mmp14* were less similar to the *Timp1* expression profile, while *Mmp2* and *Mmp12* formed a separate cluster, with the *Mmp13* expression profile being completely unlike the expression profiles of other genes (Figure 4C).

### 2.4. Silencing of Timp1 in Macrophages RAW264.7 with siRNA

**Figure 5:**
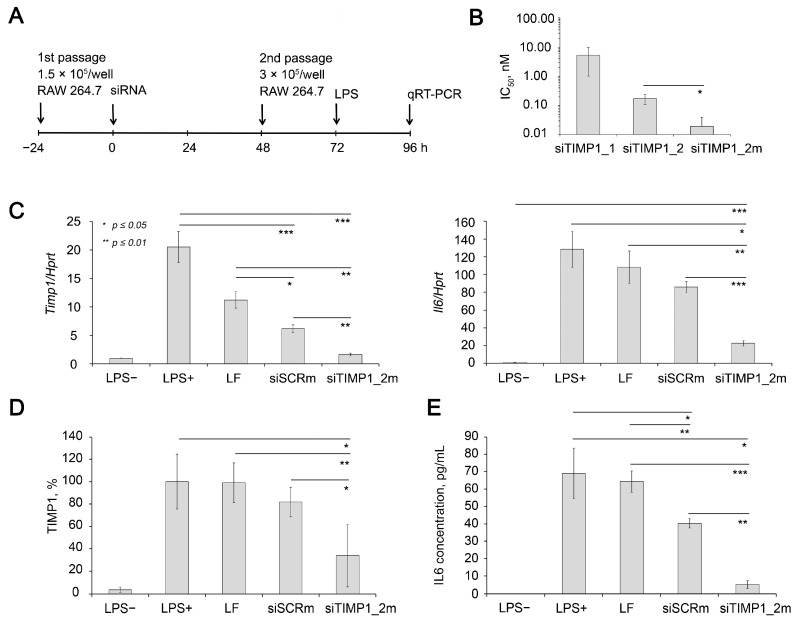
Silencing activity of anti-*Timp1* siRNA in RAW 264.7 cells. (**A**) Experimental setup. (**B**) Calculated IC_50_ of silencing activity of anti-*Timp1* siRNAs transfected using Lipofectamine 2000 in RAW 264.7 cells. The efficiency of *Timp1* silencing was compared to the untreated cells. Four independent samples were measured in triplicates for each point. (**C**) Relative mRNA levels of *Timp1* and *Il6* in RAW 264.7 cells after LPS stimulation, transfection was carried out with Lipofectamine 2000. The level in control, non-treated cells was set as 1. The data represent the mean ± standard deviation from six or seven independent experiments analyzed in triplicates. (**D**) Relative TIMP1 protein level in RAW 264.7 cells 16 h after LPS stimulation, transfection was carried out with Lipofectamine 2000 4 days before measurement. The data represent the mean ± SD from three experiments. Representative Western blot image is presented at Supplementary Figure S3. Three independent samples were analyzed for each point. (**E**) Relative IL6 protein level in the culture medium of RAW 264.7 cells 16 h after LPS stimulation; transfection was carried out with Lipofectamine 2000 4 days before LPS stimulation. The data represent the mean ± SD from three experiments. Three independent samples were measured in duplicates for each point. * *p* ≤ 0.05, ** *p* ≤ 0.01, *** *p* ≤ 0.001, Student’s two-tailed unpaired *t*-test.

To confirm the role of *Timp1* as one of the master regulators of inflammation and a potential target in anti-inflammatory therapy, we silenced *Timp1* with anti-*TIMP1* siRNA in macrophages RAW264.7 (Figure 5), since according to our data (Figure 2) *Timp1* is up-regulated in these cells in response to LPS treatment.

We compared the efficiency of two siRNA sequences containing 2′-O-methyl (2′-OMe) modifications at nuclease-resistant sites targeted to 204-224 nt or 500-520 nt of *Timp1* mRNA (NM_001044384.1, siTIMP1_1 or siTIMP1_2, respectively) (Table 1). Data showed that calculated IC_50_ (half-maximal inhibitory concentration) for *Timp1* mRNA were 5.37 and 0.18 nM for siTIMP1_1 and siTIMP1_2, respectively (Figure 5B). Previously, it was shown that the effectiveness of siRNA silencing activity can be improved by a more intense pattern of chemical modifications [89]. Therefore, we compared the efficiency of gene silencing of partly modified siTIMP1_2 and fully modified siTIMP1_2m (Table 1). It was shown that siTIMP1_2m has IC_50_ by an order of magnitude less than parent siTIMP1_2 (0.18 and 0.02 nM for siTIMP1_2 and siTIMP1_2m, respectively (Figure 5B). siTIMP1_2m silenced *Timp1* mRNA by 90% compared to LPS treated cells, which corresponds to *Timp1* mRNA level in unstimulated cells (LPS-).

It should be noted that a slight decrease in the expression level of the *Timp1* was also observed, when cells were exposed to Lipofectamin 2000 (LF) and to siSCR with a control sequence complexed with LF. However, the levels of these effects are significantly lower than those under the action of a specific siRNA. (Figure 5C). Possible explanation of down-regulation of *Timp1* with siSCR can be found in the nonspecific effects induced by siRNAs, including non-targeted hybridization, aptamer effect, binding to cellular proteins, translational silencing through miRNA-like mode of action or immunostimulation [90]. Western blot data (Figure 5D and Supplementary Figure S3) showed that siTIMP1_2m reduced the level of TIMP1 protein by 66% compared with LPS-treated cells, while siSCR only decreased its level by 18%; the effect of the transfection agent on the protein level was insignificant.

Since RAW 264.7 cell line is a classical cellular model of inflammation characterized by significant up-regulation of several cytokines including *Il6* in response to LPS treatment [91,92,93], we evaluated *Il6* mRNA level in cells with silenced *Timp1* to clarify the issue of how the expression of *Timp1* and *Il6* are related. The data showed that in cells with silenced *Timp1*, the activation of *Il6* under the action of LPS is significantly reduced and its level is 83% lower than in untreated cells stimulated by LPS (Figure 5C). It should be noted that cationic liposomes and their complex with control siRNA (siSCR) also somewhat reduce the level *Il6* expression, but less significantly than the level of *Timp1*. The data show that the decrease in *Il6* expression was 33 and 15.6% for siSCR/2X3-DOPE and 2X3-DOPE alone, respectively. This may be due to the known and the previously described immunostimulatory effect of cationic liposomes. Simultaneously, the level of *Il6* in cells with silenced *Timp1* was 74% lower than in cells treated with siSCR/2X3-DOPE. Determination of the level of IL6 using ELISA (Figure 5E) in a culture medium of LPS stimulated RAW 264.7 cells showed complementary data: silencing of the TIMP1 gene reduces the level of IL6 by 93%, while siSCR only reduces it by 42%; the influence of the transfer agent is also insignificant. Thus, the obtained data confidently proves that in RAW 264.7 cells, *Timp1* regulates the *Il6* expression, and not vice versa. It should be considered that *Timp1* is obviously only one of the regulators of *Il6* expression in this system since almost complete normalization of the *Timp1* expression by siTimp1_2m does not lead to the same effect for *Il6*: its level is 22.48 times higher than the level in unstimulated cells, which is significantly lower than the level achieved without *Timp1* silencing which exceeds the level of unstimulated cells by 128.4-fold. Thus, here for the first time we showed the possibility of restoring the IL6 expression level activated by LPS by using anti-Timp1 siRNA-mediated *Timp1* silencing.

### 2.5. Dynamics of Timp1 Expression and Silencing in the Lung Tissue of Mice during ALI Development

**Figure 6:**
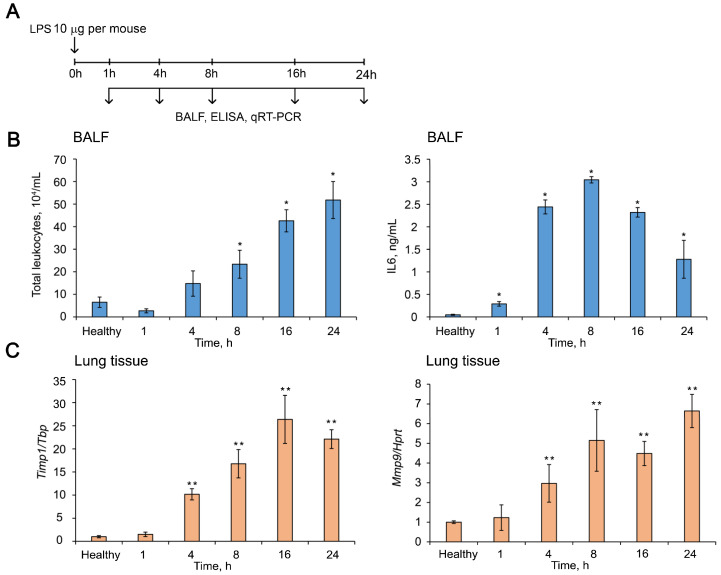
Dynamics of ALI development in mice after LPS challenge. (**A**) Experimental setup. Mice (n = 6) were intranasally (i.n.) challenged with LPS (10 µg per mouse) followed by collecting bronchial alveolar lavage (BAL) fluid and lung tissue at 1, 4, 8, 16, and 24 h after induction. (**B**) Total leukocytes counts (**left panel**) and the level of pro-inflammatory cytokine IL6 measured by ELISA (**right panel**) in the BAL fluid of LPS-challenged mice. Four BAL samples from each experimental group were analyzed. (**C**) The expression levels of *Timp1* (**left panel**) and *Mmp9* (**right panel**) in the lung tissue of LPS-challenged mice measured by RT-qPCR. Expression levels were normalized to the expression level of *Tbp* (for *Timp1*) and *Hprt* (for *Mmp9*) used as reference genes. The level in control, non-treated cells was set as 1. Three to five samples from each experimental group were analyzed in triplicate. Three to five mice from each experimental group with two lung tissue samples from each mouse were analyzed in triplicate. The data are shown as mean ± standard deviation. * *p* ≤ 0.05, ** *p* ≤ 0.01, Mann–Whitney U-test.

In order to evaluate the kinetics of *Timp1* gene expression changes in the lung tissue during ALI, the murine model of LPS-induced ALI was used. Mice were subjected to intranasal (i.n.) administration of LPS (10 µg per mouse) followed by collecting bronchial alveolar lavage (BAL) fluid and lung tissue at 1, 4, 8, 16, and 24 h after induction (Figure 6A). Total leukocyte counting, ELISA and RT-qPCR analysis were performed (Figure 6).

The development of LPS-induced ALI was accompanied by an increase in the number of total leukocytes and the level of pro-inflammatory cytokine IL6 in the BAL fluid as well as by the enhancement of expression levels of *Timp1* and related *Mmps* in the lung tissue (Figure 6B,C and Supplementary Figure S1). The number of total leukocytes in the BAL fluid of LPS-challenged mice was increased by 8-fold compared to the healthy animals, reaching the peak 24 h after induction (Figure 6B, left panel). IL6 dynamic was characterized by maximum values 8 h after induction, which were 60-fold higher than the healthy subjects (Figure 6B, right panel).

The expression level of *Timp1* mRNA in the lung tissue of mice with ALI was 26-fold higher than in healthy lungs, reaching a maximum 16 h after induction with subsequent decline (Figure 6C, left panel). The dynamics of the expression levels of related Mmps in the inflamed lungs was quite different (Supplementary Figure S1); however, the expression of one of the most important Mmps–Mmp9 was increased by 5-fold compared to healthy control by 8 h after induction and remained at this level until 24 h, the end of the experiment (Figure 6C, right panel). Thus, the time point 16 h after LPS administration is characterized by the most pronounced changes in both the expression of the potential target and markers of inflammation; therefore, we used this time point in the following experiments.

**Figure 7:**
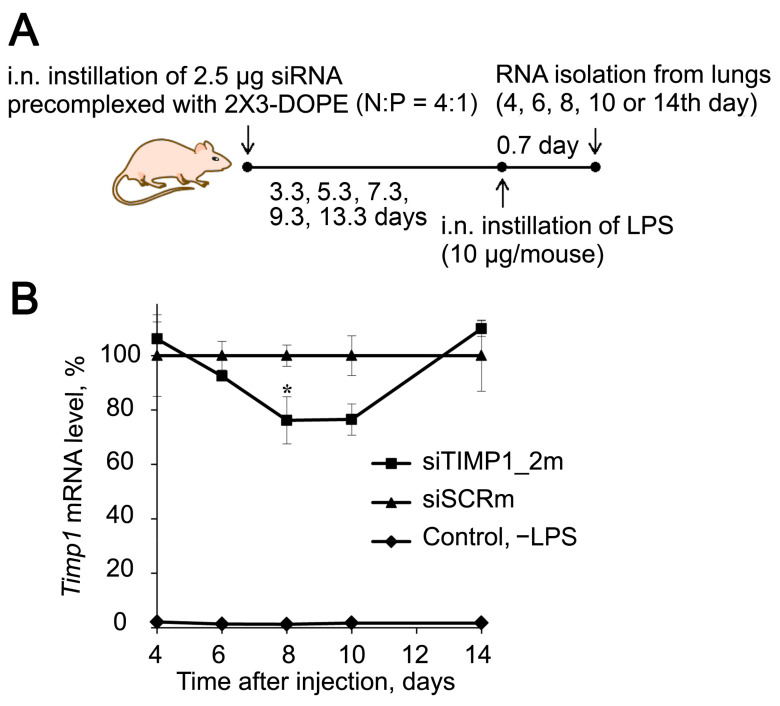
Silencing activity of anti-*Timp1* siRNA in vivo. (**A**) Experimental setup. siTIMP1 or siSCR pre-complexed with cationic liposomes 2X3-DOPE were i.n. administered to mice, then at different time points mice (n = 3) were challenged with LPS and 16 h after induction, lungs were collected for isolation of total RNA. (**B**) Kinetic of relative *Timp1* mRNA level in BALB/c mice receiving siTIMP1_2m or siSCR before LPS challenge. Control—no LPS. Mean values (±SEM) and statistical significance of differences (difference from the siSCR, * *p* < 0.05), calculated from the results of two independent experiments, are shown, Mann–Whitney U-test.

Then, we studied the kinetics of the changes in the *Timp1* mRNA level in the lungs after i.n. administration of siTIMP1_2m pre-complexed with liposomes 2X3-DOPE followed by LPS challenge to validate the silencing activity of the selected siRNA in vivo (Figure 7A). The cationic liposomes 2X3-DOPE was chosen as the transfection agent for in vivo experiments, since its high transfection efficiency and low toxicity have been previously shown [94,95]. Time points of 4–14 days after siRNA administration and before LPS challenge were chosen based on the literature data and our previously obtained results [96]. As depicted in Figure 5B, siTIMP1_2m decreased the level of *Timp1* by 25% 8–10 days after i.n. administration, then to 14th day the mRNA level restored to the level in siSCR-treated mice (Figure 7B). Based on these data, a time point of 8 days was chosen to study the effect of *Timp1* mRNA silencing on the development of ALI in a mouse model.

### 2.6. Anti-Timp1 siRNA Effectively Suppress LPS-induced Lung Inflammation In Vivo

**Figure 8:**
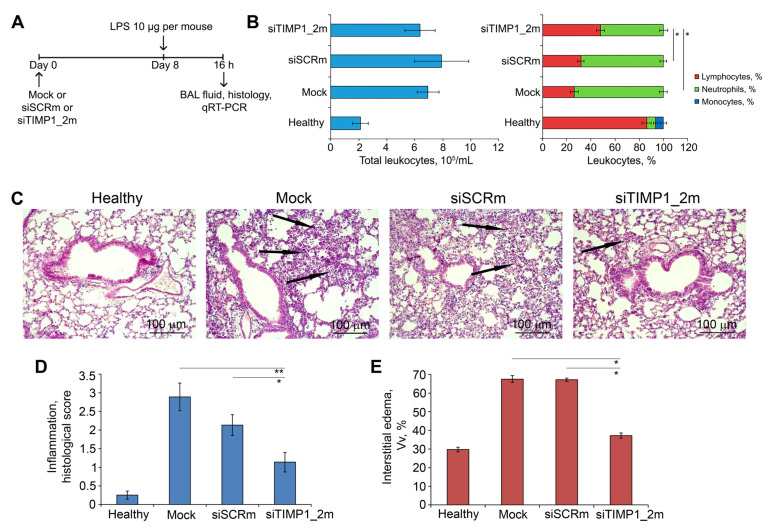
siTIMP1_2m effectively suppresses LPS-induced lung inflammation in vivo. (**A**) Experimental setup. Acute lung injury (ALI) was induced in mice (n = 6) by intranasal (i.n.) instillations of LPS (10 µg per mouse). Mice were pretreated with siTIMP1_2m administered i.n. 8 days before ALI induction. Mock and siSCR were used as controls. Mice were sacrificed 16 h after LPS challenge followed by collection of bronchoalveolar lavage (BAL) fluid and the lung tissue for subsequent analysis. (**B**) Total (**left**) and differential (**right**) leukocyte counts in the BAL fluid of healthy and LPS-challenged mice without treatment and after siTIMP1 administration. Four BAL samples from each experimental group were analyzed. (**C**) Representative histological images of lung tissue of ALI mice without treatment and after siTIMP1_2m administration. Haematoxylin and eosin staining. Original magnification ×200. Black arrows indicate inflammatory infiltration in the lung tissue. (**D**,**E**) The intensity of inflammatory infiltration quantified by the histological scoring system (**D**) and the volume density of interstitial edema (**E**) in the lung tissue of LPS-challenged mice without treatment and after siTIMP1 administration. The inflammatory scores and interstitial edema were calculated in 5 random fields in each lung sample, forming 30 random fields from each experimental group. Data are presented as mean ± standard deviation. * *p* ≤ 0.05, ** *p* ≤ 0.01, Mann–Whitney U-test.

Experimental setup is depicted in Figure 8A. Mice were pretreated with siTIMP1_2m/2X3-DOPE complexes via i.n. instillations 8 days before ALI induction. ALI was induced in mice by i.n. administration of LPS (10 µg per mouse). Mice pretreated with siSCRm/2X3-DOPE and 2X3-DOPE only (Mock) were used as controls. Animals were sacrificed 16 h after ALI induction bronchoalveolar lavage (BAL) fluid and lung tissue samples were collected for subsequent analysis (Figure 8A).

The development of LPS-induced ALI was accompanied by significant inflammatory changes in the respiratory system of mice. Analysis of BAL fluid of LPS-challenged mice treated with Mock and siSCRm revealed a 3.2- and 3.7-fold increase in the number of total leukocytes predominately due to neutrophils compared with healthy animals (Figure 8B, left panel). Administration of siTIMP1_2m led to the inhibition of the leukocyte migration into the BAL fluid expressed in a 1.4- and 1.3-fold decrease in the fraction of neutrophils compared with Mock- and siSCR-treated animals (Figure 8B, right panel).

Histological analysis of lung tissue of LPS-challenged and Mock-treated mice revealed marked inflammatory, circulatory and destructive changes in the lung tissue, represented by the infiltration of lung tissue with granulocytes, venous plethora and hemorrhages, desquamation of the bronchial and alveolar epithelium (Figure 8C). Treatment of ALI mice with siTIMP1_2m caused significant suppression of inflammatory, circulatory, and destructive changes in their respiratory system: only residual inflammatory cells in the interalveolar space were observed (Figure 8C). Calculation of the histological score revealed a 2.5-fold decrease in the intensity of inflammatory changes in the lung tissue of LPS-challenged mice after siTimp1_2m administration (Figure 8D). Administration of siSCR slightly inhibited the LPS-induced ALI causing a 1.4-fold decrease in the intensity of inflammation in the lungs of LPS-challenged mice (Figure 8D). Evaluation of LPS-driven interstitial edema by the calculation of the volume density (Vv, %) of inter-alveolar septa revealed a 2.3-fold increase of this parameter in the lung tissue of ALI mice after Mock and siSCRm treatment (Figure 8E and Supplementary Figure S4). Administration of siTIMP1_2m led to a 1.8-fold decrease in the thickness of the inter-alveolar septa compared with Mock- and siSCRm-treated animals bringing this parameter almost to the values of healthy animals (Figure 8E and Supplementary Figure S4). Thus, the obtained data indicate that anti-Timp1-siRNA reduces the severity of LPS-induced acute lung injury.

## 3. Discussion

Acute lung injury is a complex cascade process that develops in response to various damaging factors, which can lead to acute respiratory distress syndrome. Currently, there are no specific markers for the course of ALI, which complicates the prognosis; moreover, the absence of etiotropic treatment does not allow obtaining a significant therapeutic effect and improve the survival of patients [20]. Therefore, the search for molecular markers, potential therapeutic targets, and treatments is essential.

We selected nine candidate genes that are closely associated with inflammation according to the available literature data and bioinformatics. We screened these genes in five cell lines of different embryonic origins (endo, ecto, and mesoderm) to better understand their response to pro-inflammatory stimuli (LPS) and to assess their potential relevance as a molecular marker/target for ALI treatment.

As expected, RAW264.7 and J774 macrophages showed the best inflammatory response compared to the other cell lines. We observed the highest production of the pro-inflammatory cytokines IL6 and TNF-α in these cells. It is notable that macrophages are among the most important cells involved in the pathophysiology of inflammation [97]. Among the genes studied, only *Timp1* and *Mmp9* demonstrated significant expression activation in response to LPS stimulation. Matrix metalloproteinases and their master regulators are critical to the pathophysiology of ARDS and may be useful in predicting the course of inflammation [98]. After the correlation analysis, *Timp1* turned out to be the best candidate target for further study among other genes. The fact that in our study *Timp1* demonstrates a central role in the inflammatory process is consistent with data on its key role in the pathophysiology of inflammation [52,53,54]. 

Functional analysis of *Timp1* and its first neighbors in gene networks with subsequent validation on the murine model of LPS-induced acute lung injury at different time points (Figure 4) confirmed close involvement of *Timp1* gene and its first neighbors both in acute inflammation and its long-term consequences such as fibrosis development and tumor transformation. The opposite involvement of *Timp1* and some *Mmp*s in the extracellular matrix regulation and pointing the necessity for a more thorough approach to their silencing using gene-targeted therapy.

Small interfering RNAs are actively used to elucidate the role of genes in the onset and development of diseases, as well as to assess their potential as targets for therapeutic drugs [99]. siRNAs aimed at suppressing the expression of the *Timp1* gene were previously used by researchers in studies related to the development of tumor and pathological processes affecting the connective tissue and extracellular matrix (ECM) [100,101,102,103,104,105,106,107]. The involvement of *Timp1* in the processes of proliferation, migration, and apoptosis has been shown for tumors of various histogenesis [100,101,102,103]. Thus, it was shown that the inhibition of the expression of this gene in diffuse large B-cell lymphoma (DLBCL) cells by siRNA decreases the expression of Wnt target genes associated with DLBCL progression, inhibits their migration, and regulates the Wnt signaling pathway [100]. Blocking *Timp1* expression with siRNA increased the sensitivity of pancreatic ductal adenocarcinoma cells to gemcitabine and reversed their resistance to chemotherapy; the combined action of shTIMP1 therapy and gemcitabine induced apoptosis of tumor cells [101]. The suppression of *Timp1* inhibited melanoma growth and lung colonization, as well as reduced resistance to dacarbazine [102].

The role of inducible *Timp1* expression in the development of fibrosis has been investigated in several models [104,105,106]. Liver cell apoptosis induced by various damaging agents (GCDC, LPS, alcohol) activates *Timp1* and induces fibrosis, while treatment with TIMP1-siRNA inhibits the expression of fibrosis-related genes *aSMA*, *CTGF*, and *TGFb2r* and reduces the amount of fibrotically altered liver tissue [104]. The role of increased expression of the *Timp1* gene was revealed not only in the prevention of apoptosis of hepatic stellate cells (HSC), which play a key role in the development of liver fibrosis, but also its Act-dependent mitogenic effect on these cells [105]. *Timp1* expression has the opposite effect on human mesenchymal stem cells (hMSCs): silencing of *Timp1* revealed that endogenous TIMP1 suppresses the proliferation, metabolic activity, and osteogenic differentiation capacity of hMSCs acting via a Wnt/β-catenin signaling pathway [106]. Considering these data together, it can be concluded that the inhibition of *Timp1* expression may contribute to the resolution of pathological conditions associated with tissue damage and excessive extracellular matrix deposition [107] in various ways. Efforts to develop siRNAs and means for their delivery to the lungs for the treatment and prevention of pulmonary diseases, including acute lung injury, have been undertaken by many research groups [36,108]. As targets, genes such as *Tnf-α* [109], *PD-L1* [110], *Rip2* [111], *Myd88* [112], *Paxillin* [113] have been used, but despite much evidence that its overexpression is observed in inflammatory processes and lung damage, *Timp1* has never been considered a promising target for this purpose.

We prepared a chemically modified siTimp1_2 capable of downregulating *Timp1* expression in vitro and in vivo when used along with cationic liposomes. It should be noted that silencing in RAW264.7 cells under the action of siTimp1_2 in vitro occurs much more efficiently compared with the action of the same siRNA in lung tissue in vivo (74% and 24% silencing compared to siSCR, respectively) (Figure 5C and Figure 7B). These differences in siRNA performance may be influenced by three main factors. The first factor is the difference in levels of *Timp1* expression between LPS challenged mice and stimulated RAW264.7 cells (a 61.5-fold increase in the lung and 20.5-fold increase in the cultured cells compared to the control). In contrast to the in vitro model, where LPS activates RAW264.7 macrophages, releasing pro-inflammatory cytokines and chemokines, in vivo LPS activates not only macrophages but also induces the infiltration of monocytes and neutrophils into the alveolar space, which release additional inflammatory mediators that trigger the pulmonary inflammatory cascade [38]. The second factor is the limited efficiency of siRNA delivery in vivo, due to the existence of mucous and anionic surfactant layers in the lungs, which can disrupt siRNA/cationic liposome complexes, and the presence in the lungs of macrophages that could reduce the concentration of siRNA/2X3-DOPE by phagocytosis [108]. Altogether these biological barriers could decrease the siRNA concentration in target cells. The third factor is the ribonuclease activity, which has a much greater effect on siRNA integrity in vivo than in vitro [114]. Although siRNA is pre-complexed with liposomes and all 2′OH groups are protected by 2′F or 2′O-methyl modifications from the action of endonucleases, siTimp1_2m does not contain PS modifications that significantly protect siRNA against exonucleases. It can be expected that the introduction of PS modifications into the composition of anti-Timp1 siRNA will improve its silencing activity in vivo. In this regard, the question arises whether it is necessary to strive for complete blocking of *Timp1* mRNA expression. Data from *Timp1*-deficient mice indicate that *Timp1* loss exacerbates blood–brain barrier disruption [115], exhibits increased cardiovascular permeability, and is associated with impaired skeletal muscle microvasculature [116]. In contrast to the permanent silencing of *Timp1* throughout the body, temporary silencing for several months may be promising for antifibrotic therapy in various organs [117]. Therefore, the use of a more efficient and stable anti-Timp1 siRNA and optimization of delivery methods may be a promising approach for anti-inflammatory and anti-fibrotic therapies and could be the subject of further research.

In our work, it was clearly demonstrated that even a moderate suppression of *Timp1* gene expression in the lung tissue of experimental animals leads to a significant improvement in inflammatory and destructive changes in the respiratory system of mice, presumably due to the long-term inhibitory effect of nuclease-resistant siRNA used in the current study. Thus, we can assume the effective use of such gene silencing instruments both for treating acute inflammatory changes and their long-term consequences, such as fibrosis of internal organs. Moreover, this hypothesis can be supported by the close involvement of *Timp1* and *Mmp*s as its first neighbors in the processes of ECM organization and tissue remodeling as well as participation in the pulmonary and liver fibrosis (Figure 4B). It should be noted that the effective usage of Timp1-targeted constructions, such as siRNA and shRNA, for the treatment of liver fibrosis [104,118] and other diseases accompanied by an imbalance in the synthesis/degradation of ECM components, such as keloids, was shown [119]. Additionally, *Timp1* knockdown decreases proliferation, migration and invasion of tumor cells of diverse origins [120,121,122].

Thus, the data obtained allow us to conclude that *Timp1* is a promising new molecular target for treating ALI. We show that siRNA-mediated prevention of *Timp1* activation during LPS-induced ALI significantly decreased pro-inflammatory changes in the lung tissue. Moreover, *Timp1* silencing prevents or inhibits cytokine IL6 secretion by macrophages, which significantly contribute to the reduction in inflammation, as well as indicate a potentially wider use of *Timp1* inhibitors for therapeutic purposes. All these factors point to the prospects for *Timp1* silencing as an approach to therapy of not only acute inflammatory changes in the lung tissue but also their long-term effects, such as pulmonary fibrosis, which is the goal of our further investigations.

The gene-targeted approach used in this study is up-to-date due to the resurgent wave of the COVID-19 epidemic [123], since one of the main factors threatening the health and life of patients infected with SARS-CoV-2 is acute respiratory distress syndrome (ARDS), which occurs as one of the severe manifestations/complications of COVID-19 [124]. Timp1-targeted siRNA was shown to prevent inflammatory and destructive changes in the lungs in an LPS-induced ALI model in mice, which is highly relevant to COVID-19-associated ARDS in humans [125]. Thus, the pretreatment approach effectively used in our work can be proposed for the prevention of severe lung complications of COVID-19 in clinical practice.

The main factor limiting the use of siRNAs to suppress therapeutic targets in the lungs is the lack of an effective delivery scheme. Intranasal, intratracheal, or orotracheal administration of siRNA pre-complexed with liposomes, particles, or dendrimers is used in experimental schemes for administering siRNA-based drugs to mice [126]. These schemes take into account the characteristics of the respiratory tract of the mouse, allowing for effective intranasal administration to the lungs. We used cationic liposomes 2X3-DOPE to deliver siRNA to the lungs; however, both the liposomes themselves and the TIMP1 inhibitor should be used with some caution and after a thorough safety assessment. It has been shown that intranasal siRNA delivery can be used to silence genes in the brain [127], so the possible unwanted downregulation of target gene expression in the brain should be taken into account. It is also known that cationic liposomes themselves can exhibit an immunostimulatory effect, so their combined effect with LPS or an infectious agent should be evaluated. From this point of view, a safer method of administration is inhalation or nebulization; both of these methods can be used in combination with liposomal delivery vehicles and reduce the contact of the aerosol with the olfactory epithelium, which is involved in the passage to the brain. Currently, these methods are actively used in pharmacy for the delivery of small molecule drugs and are being studied in experimental work on the delivery of siRNA to the lungs [128,129,130], which makes it possible to develop siRNA-based drugs for the treatment of lung diseases

## 4. Materials and Methods

### 4.1. Identification of Hub Genes, Their Functional Analysis and PPI Network Reconstruction

In order to reveal key genes associated with acute inflammation, gene expression profiles GSE94522 (3 healthy mice, 3 bleomycin-treated mice; lung tissue), GSE122197 (7 PBS-treated mice, 8 ovalbumin-sensitized mice; lung tissue), and GSE35609 (4 healthy mice, 5 DNBS-treated mice; colon tissue) were obtained from the Gene Expression Omnibus (GEO) database and analyzed by the GEO2R tool (pathology vs. control). The revealed DEGs (|fold change| ≥ 1.5, *p* < 0.05) were further overlapped using InteractiVenn tool [131].

Functional annotation of the revealed DEGs and reconstruction of gene association and protein–protein interaction (PPI) networks were performed using the ToppGene web tool [132] and Search Tool for the Retrieval of Interacting Genes/Genomes (STRING) database, respectively. The gene/PPI network reconstruction in STRING was performed using interactions with a confidence score > 0.7, and reconstructed protein pairs included functional relations of proteins from seven sources: text mining, experimental data, databases, co-expression data, neighborhood, gene fusion and co-occurrence. Reconstructed networks were visualized as undirected networks in Cytoscape 3.7.2. Topology analysis of the reconstructed networks was performed using the NetworkAnalyzer 3.3.2 plugin in Cytoscape. Functional enrichment analysis was performed through the ToppFun module of the ToppGene suite, using the MSigDB Biocarta (v.7.5.1), BioSystems: Reactome, KEGG, Panther DB, DisGeNET BeFree, DisGeNET Curated and Gene Ontology: biological processes databases. One gene set and default options were used in all enrichment tools: a significance threshold of 0.05 for Bonferroni adjusted *p*-value, at least five genes from the input list in the enriched category, and the whole genome as the background.

### 4.2. Cell Culture

The murine macrophage cell line RAW 264.7 was kindly provided by Professor D. Kuprash (Engelhard Institute of Molecular Biology, Moscow, Russia). J774 macrophage, Hepa 1-6 hepatocellular carcinoma, B16 melanoma, L929 fibroblast cell lines were obtained from the Russian Cell Culture Collection (Institute of Cytology, RAS, St. Petersburg, Russia). Cells were cultured in Dulbecco’s modified Eagle’s medium (DMEM) (Sigma-Aldrich Inc., St. Louis, MO, USA) supplemented with 10% (*v*/*v*) heat-inactivated fetal bovine serum (BioloT, St. Petersburg, Russia) and an antibiotic-antimycotic solution (100 U/mL penicillin, 100 µg/mL streptomycin, 0.25 µg/mL amphotericin) and incubated at 37 °C in humidified 5% CO_2_-containing air atmosphere (hereafter standard conditions).

### 4.3. LPS Treatment

Cells were plated into 6 well plates at a density of 0.3 × 10^6^ cells/well (L929) 0.5 × 10^6^ cells/well (other cell lines) and incubated for 24 h before LPS stimulation (1 µg/mL). After 3, 6, 9, 16, 20 or 24 h incubation with LPS growth media was removed and TRIzol Reagent (Ambion, Carlsbad, CA, USA) (1 mL/well) was added directly to the culture dish to lyse the cells. Cell lysates were pipetted several times to homogenize cells and frozen until RNA isolation. Samples were stored at −70 °C.

### 4.4. Mice

Female 6–8-week-old Balb/C mice (average weight 20–22 g) were obtained from the Vivarium of the Institute of Chemical Biology and Fundamental Medicine SB RAS (Novosibirsk, Russia). The mice were housed in plastic cages (6 animals per cage) under normal daylight conditions. Water and food were provided ad libitum. Experiments were carried out in accordance with the European Communities Council Directive 86/609/CEE. The experimental protocols were approved by the Committee on the Ethics of Animal Experiments of the Administration of the Siberian Branch of the Russian Academy of Sciences (Novosibirsk, Russia) (protocol No. 56 from 10 August 2019).

### 4.5. Real-Time Quantitative PCR (RT-qPCR) Assay

Total RNA was isolated from cells using TRIzol Reagent according to the manufacturer’s instructions. The RNA yield and quality were determined by measuring the absorbance at 260 nm and 280 nm using a NanoDrop spectrophotometer. cDNAs were synthesized using a RT buffer and M-MuLV-RH revertase (Biolabmix, Novosibirsk, Russia), according to the manufacturer’s instructions. Briefly, 20 μL reaction mixtures containing 4 μL 5 × RT buffer mix (contains dNTP), 20 pmol dT15 primer, 1 μg of total RNA, 50 U of M-MuLV Reverse Transcriptase were incubated for 60 min at 42 °C followed by heating the solution at 70 °C for 10 min. RT-qPCR was carried out using HS-qPCR (×2) master mix (Biolabmix, Novosibirsk, Russia) according to the manufacturer’s instructions. Briefly, 25 μL reaction mixtures contained 12.5 μL HS-qPCR (×2) master mix, 10 pmol of each primer, 1 μL of cDNA. Amplification was performed using temperature conditions: (1) 94 °C, 5 min; (2) 94 °C, 10 s; 60 °C, 30 s (50 cycles). The sequences of primers and probes for *Tnf-α*, *Il6*, *C3*, *Serpina3a*, *Dap12*, *Adam8*, *Timp1*, *Trem2*, *Mmp9*, and *Hprt* are listed in Table 2. The relative level of gene expression was calculated using the Bio-Rad CFX software (Bio-Rad Laboratories Inc., Hercules, CA, USA). *Hprt* was used as the reference gene.

### 4.6. ELISA

Supernatants from RAW 264.7 cells were collected and analyzed for IL6 production using ELISA (Thermo Fisher Scientific, Vienna, Austria) according to the manufacturer’s protocol (detection limits 8–200 pg/mL). The absorbance was measured at 450 nm using a Multiscan RC plate reader (Thermo LabSystems, Helsinki, Finland). The experiment was repeated twice with three parallel measurements each.

### 4.7. Synthesis of siRNAs and Duplex Annealing

The 2′-F and 2′-OMe-modified sense and antisense strands of siRNAs were synthesized on an automatic ASM-800 DNA/RNA synthesizer (Biosset, Novosibirsk, Russia) using 2′-F-ribo- and 2′-O-methylribo β-cyanoethyl phosphoramidites (Glen Research, Brook Park, OH, USA). Oligoribonucleotides were purified by 15% polyacrylamide/8M urea gel electrophoresis (PAGE) and isolated as sodium salts. The purified oligoribonucleotides were characterized by MALDI-TOF mass spectrometry on a PEFLEX III spectrometer (Bruker Daltonics, Bremen, Germany). The siRNA sequences are listed in Table 1. siSCR lacking significant homology to any known mouse, rat, or human mRNA sequence was used as a control. siRNAs were obtained by annealing 200 mM sense and antisense strands in the buffer containing 30 mM HEPES-KOH (pH 7.4), 100 mM sodium acetate, and 2 mM magnesium acetate, and subsequently stored at −20 °C.

### 4.8. Transfection of siRNA

One day before the experiment, RAW 264.7 cells in the exponential phase of growth were plated in 6-well plates at a density of 1.5 × 10^5^ cells/well. After 24 h, the growth medium was replaced by fresh serum-free DMEM (2 mL/well). The cells were transfected with siRNAs (0.1–100 nM) in 500 µL of Opti-Mem using Lipofectamine 2000 (Invitrogen, Waltham, MA, USA) according to the manufacturer’s protocol (10 µL of Lipofectamine 2000 per well). Two days after transfection, cells were replated to prevent overgrowth. Then, three days after transfection, LPS (final concentration 1 nM) was added to the cells. Four days after transfection, total RNA was isolated from the cells and RT-qPCR was performed as described above.

### 4.9. Western Blotting

RAW 264.7 cells were lysed in a 24 well plate in 60 μL of sample buffer (Sigma-Aldrich, St. Louis, MO, USA) after transfection and LPS treatment (described in Section 4.8). Of each sample, 15 μL was loaded on a 10% SDS/polyacrylamide gel and then separated at 15 mA for 1 h. The proteins were transferred from PAAG to PVDF membrane (Millipore, Burlington, MA, USA) using a Bio-Rad Criterion Blotter (Bio-Rad Laboratories Inc., Hercules, CA, USA), and the membrane was then blocked overnight in 2% non-fat dried milk in 0.05 M Tris–HCl, 0.15 M NaCl, and 0.1% Tween-20 pH 7.5. The membranes were incubated with monoclonal anti-TIMP1 (102D1, dilution 1:50, Thermo Fisher Scientific, Waltham, MA, USA) or anti-GAPDH antibodies (A19056, dilution 1:5000, A19056, ABclonal, Wuhan, China) overnight. After the membranes were washed in 0.05 M Tris–HCl, 0.15 M NaCl, 0.1% Tween-20 pH 7.5, they were subsequently incubated for 1 h with secondary goat anti-mouse (A9917, dilution: 1:5000, Sigma Aldrich, St. Louis, MO, USA) or goat anti-rabbit (AS014, dilution: 1:2000, ABclonal, Wuhan, China) antibodies conjugated with horseradish peroxidase. The visualization was performed using a Western Blotting Chemiluminescent Reagent Kit (Abcam, Waltham, MA, USA). Data were analyzed using iBright Analysis Software version 5.1.0 (Thermo Fisher Scientific, Waltham, MA, USA).

### 4.10. In Vivo Model of Acute Lung Injury (ALI)

ALI was induced in Balb/C mice by intranasal (i.n.) instillation of 10 µg LPS in 50 µL of saline per mice under isoflurane anesthesia. In the kinetics studies, mice (4 per group) were sacrificed 1, 4, 8, 16, and 24 h after ALI induction followed by collection of lung tissue and bronchoalveolar lavage (BAL) fluid for subsequent analysis.

In the studies of the anti-inflammatory potential of siTimp1, the mice (6 per group) received 2.5 µg siRNA pre-complexed with cationic liposomes 2X3-DOPE 8 days before the ALI induction. The mice received 2X3-DOPE alone (Mock) or siScr/2X3-DOPE complexes were used as controls. The mice were sacrificed 16 h after ALI induction, and the lung tissues and BAL fluid were collected for subsequent analysis.

### 4.11. Silencing of Timp1 in Mice with siRNA

Liposomal delivery system based on polycationic amphiphile 1,26-bis (cholest-5-en-3-yloxycarbonylamino)-7,11,16,20-tetraazagehexacosane tetrahydrochloride (2X3) and lipid-helper dioleoylphosphatidylethanolamine (DOPE), which efficiently delivers nucleic acids in vitro and in vivo was used for in vivo experiments [95,133]. Anti-*Timp1* siRNA (2.5 µg) pre-complexed with cationic liposome 2X3-DOPE (N/P 4/1) in 100 µL Opti-Mem was intranasally instilled to Balb/C mice (3 per group), then in 80, 128, 176, 224 and 320 h mice were challenged intranasally with 10 µg LPS (055:B5, Sigma-Aldrich, St. Louis, MO, USA) in 50 µL of saline. Sixteen hours after the challenge, the mice were sacrificed, the total RNA was isolated from the lungs and the levels of *Timp1* mRNA were measured on 4, 6, 8, 10 or 14th day after siRNA administration by RT-qPCR, as described above.

### 4.12. Bronchoalveolar Lavage (BAL) Fluid Analysis

The lungs were lavaged with 1 mL ice-cold phosphate-buffered saline (PBS). The collected BAL fluid was centrifuged at 1500 rpm for 10 min at 4 °C. The supernatants were used for subsequent protein study. The cell pellets were resuspended in 50 μL of PBS, and total leukocyte counts were performed using a Neubauer chamber by optical microscopy after diluting in Türk solution (1:20). The differential leukocyte counts (subpopulations of granulocytes, lymphocytes, and monocytes) were calculated in BAL fluid after azur-eosin staining by Romanowsky–Giemsa by optical microscopy.

### 4.13. Histology

For the histological study, lung specimens were fixed in 10% neutral-buffered formalin (BioVitrum, Moscow, Russia), dehydrated in ascending ethanols and xylols and embedded in HISTOMIX paraffin (BioVitrum, Moscow, Russia). The paraffin sections (5 μm) were sliced on a Microm HM 355S microtome (Thermo Fisher Scientific, Waltham, MA, USA) and stained with haematoxylin and eosin. Images were examined and scanned using an Axiostar Plus microscope equipped with an Axiocam MRc5 digital camera (Zeiss, Oberkochen, Germany) at magnifications of ×200.

The histological quantitation of the lung inflammation severity was performed using a scoring system comprising the intensity of inflammatory infiltration in the lung tissue: 0—none, 1—slight, 2—moderate, 3—severe, 4—total. The scores were calculated of 5 random fields in each lung sample, forming 30 random fields for each group of mice in total. Morphometric analysis of lung tissue was performed using counting grid consisting of 100 testing points in a testing area equal to 3.2 × 10^6^ μm^2^ and included evaluation of the volume densities (Vv, %) of inter-alveolar septa outside the foci of inflammatory infiltration reflecting inter-alveolar septa thickness and consequently interstitial edema. Five random fields were studied in each lung specimen forming 30 random fields for each group of mice in total. Histological scoring and morphometry were performed by two blinded pathologists independently.

### 4.14. Statistical Analyses

The variables were expressed as the mean ± standard deviation (SD). For the in vitro experiments, the data were analyzed with Student’s two-tailed un-paired *t*-test. For the in vivo experiments, the data were analyzed with Mann–Whitney U-test. The statistical package STATISTICA version 10.0 has was for analysis.

## Figures and Tables

**Table 1:** Sequences of siRNA targeted *Timp1* used in the study.

Designation ^1^		Sequence ^2^
siTIMP1_1	S	CUUmGUUmGCUmAUCmACUmGAUmAGC
	AS	UmAUCmAGUmGAUmAGCmAACmAAGAG
siTIMP1_2	S	GCCUmAAGGAACGGAAAUUUCG
	AS	AAAUUUCCGUUCCUUmAGGCGG
siTIMP1_2m	S	GmCmCmUmAmAmGfGmAfAfCfGmGmAmAmAmUmUmUmCmGm
	AS	AmAfAmUmUmUfCmCmGmUmUmCmCmUfUmAfGmGmCmGmGm
siSCRm	S	CmCmAmCmUmAmCfAmUfAfCfGmAmGmAmCmUmUmGmUmUm
	AS	CmAfAmGmUmCfUmCmGmUmAmUmGmUfAmGfUmGmGmUmUm

^1^ S—Sense, AS—antisense strands of siRNA. ^2^ siRNA are presented in 5′–3′ direction, Am, Um, Gm, Cm—2′-OMe modifications; Af, Gf, Uf, Cf—2′-F modifications.

**Table 2:** Primers used for RT-qPCR.

Gene	Primer/Probe	Sequence 5′–3′
*Tnf-α*	probe	((5,6)-FAM)-CACAGAAAGCATGATCCGCGACG–BHQ1
	forward	CCCTCCAGAAAAGACACCATG
	reverse	GCCACAAGCAGGAATGAGAAG
*Il6*	probe	((5,6)-FAM)-TTGTCACCAGCATCAGTCCCAAGAA-BHQ1
	forward	AAACCGCTATGAAGTTCCTCTC
	reverse	GTGGTATCCTCTGTGAAGTCTC
*C3*	probe	((5,6)-FAM)-ACACCCTGATTGGAGCTAGTGGC-BHQ1
	forward	GTTTATTCCTTCATTTCGCCTGG
	reverse	GATGGTTATCTCTTGGGTCACC
*Serpina3a*	probe	((5,6)-FAM)-ACTGTGGATGGTCTGTGTCAGGC-BHQ1
	forward	GGCTTCTATCCTCTGATTGGC
	reverse	CCCAGGAATATGTGCTAGTGATG
*Dap12*	probe	((5,6)-FAM)-CCTTCCGCTGTCCCTTGACCTC-BHQ1
	forward	GGTGACTTGGTGTTGACTCTG
	reverse	GACCCTGAAGCTCCTGATAAG
*Adam8*	probe	((5,6)-FAM)-TCATCTGATACATCTGCCAGCCGC-BHQ1
	forward	TATGCAACCACAAGAGGGAG
	reverse	ACCAAGACCACAACCACAC
*Timp1*	probe	((5,6)-FAM)-ACTCACTGTTTGTGGACGGATCAGG-BHQ1
	forward	CTCAAAGACCTATAGTGCTGGC
	reverse	CAAAGTGACGGCTCTGGTAG
*Trem2*	probe	((5,6)-FAM)-ATCTTGCACAAGGTCCCCTCCG-BHQ1
	forward	GCTTGGTCATCTCTTTTCTGC
	reverse	GTTGAGGGCTTGGGACAG
*Mmp9*	probe	((5,6)-FAM)-TAGCGGTACAAGTATGCCTCTGCC-BHQ1
	forward	ACCTGAAAACCTCCAACCTC
	reverse	TCGAATGGCCTTTAGTGTCTG
*Hprt*	probe	((5,6)-ROX)-CTTGCTGGTGAAAAGGACCTCTCGAA-BHQ2
	forward	CCCCAAAATGGTTAAGGTTGC
	reverse	AACAAAGTCTGGCCTGTATCC

## Data Availability

Not applicable.

## Supplementary Materials

The following supporting information can be downloaded at: https://www.mdpi.com/article/10.3390/ijms24021641/s1.
