# Supplementary material for: siRNA-Mediated Timp1 Silencing Inhibited the Inflammatory Phenotype during Acute Lung Injury
Source: Int J Mol Sci. 2023 Jan 13;24(2):1641. doi: 10.3390/ijms24021641 (PMC9865963; doi:10.3390/ijms24021641)
Supplement: Supplementary file 1 [file ijms-24-01641-s001.zip › ijms-2089117-supplementary.pdf]

Supplementary data.

Table S1. Relative (LPS/Control) levels of *Il6*, *Tnf-α*, *Timp1*, *Adam8*, *Dap12*, *C3*, *Serpina3,* and *Mmp9* mRNA in different cell lines stimulated by LPS measured by RT-qPCR and normalized to the level of *Hprt* mRNA. N/A: data not available. * p≤0.05, ** p≤0.01, *** p≤0.001.

Gene	Time, h / Cell Line	3	6	9	16	20	24
Il6	**RAW 264.7**	2,44±0,42	8,98±3,83	5,31±4,88	64,76±47,56*	162,47±54,22*	91,20±35,95**
	**J774**	1307±365**	4673±1918***	N/A	1258±449**	1667±511**	2236±574**
	**Hepa 1-6**	6,08±4,99	2,06±0,83	3,51±1,21	4,31±2,50	3,87±2,25	N/A
	**B16**	9,09±7,83	7,28±2,46	N/A	2,11±0,84	5,76±2,95	1,80±0,88
	**L929**	1,10±0,50	0,64±0,20	N/A	0,36±0,09	1,42±0,70	0,40±0,15
Tnf-α	**RAW 264.7**	1,76±0,46	1,84±0,28	1,12±0,18	0,83±0,11	1,80±0,20	1,53±0,33
	**J774**	13,20±4,90*	11,12±9,28	N/A	1,85±1,06	4,68±0,64	2,48±1,70
	**Hepa 1-6**	2,08±1,51	2,25±1,49	1,79±0,28	6,61±4,48	2,73±2,02	N/A
	**L929**	N/A	1,24±0,63	N/A	0,38±0,13	2,19±1,18	0,82±0,45
Timp1	**RAW 264.7**	2,60±1,00	1,03±0,06	1,51±0,38	5,56±1,88*	4,25±0,62*	2,88±0,62
	**J774**	1,18±0,31	2,18±0,99	N/A	1,76±0,64	1,38±0,33	2,33±0,79
	**Hepa 1-6**	1,46±0,35	1,93±0,35	1,55±0,15	1,70±0,14	1,80±0,22	1,73±1,08
	**B16**	1,04±0,79	1,03±0,25	N/A	1,35±0,33	1,61±0,30	1,72±0,44
	**L929**	1,07±0,19	0,80±0,14	N/A	1,13±0,44	1,31±0,24	1,47±0,46
Adam8	**RAW 264.7**	1,06±0,39	0,85±0,33	0,57±0,15	1,54±0,24	1,59±0,23	1,77±0,56
	**J774**	1,16±0,24	2,93±0,89	N/A	1,75±0,60	5,41±0,94	6,47±1,38
	**B16**	1,05±0,36	3,54±1,39	N/A	0,47±0,19	1,26±0,60	0,76±0,62
Dap12	**RAW 264.7**	1,02±0,13	0,90±0,14	0,88±0,10	0,82±0,10	0,83±0,14	0,87±0,16
	**J774**	1,19±0,60	0,86±0,26	N/A	1,22±0,43	0,83±0,21	0,86±0,27
C3	**Hepa 1-6**	4,71±1,35**	5,98±1,24*	3,32±0,59*	5,41±1,25*	16,59±3,3*	3,32±1,25*
	**L929**	1,78±1,39	0,93±0,48	N/A	1,58±0,32	0,70±0,16	1,19±0,19
Serpina3	**J774**	0,74±0,34	1,25±0,46	N/A	0,81±0,21	3,32±0,85	0,40±0,15
	**Hepa 1-6**	0,86±0,55	1,62±1,12	1,74±0,59	6,73±4,05	3,08±2,45	N/A
	**B16**	3,25±2,66	0,63±0,32	N/A	0,13±0,11	1,03±0,54	0,20±0,19
	**L929**	0,87±0,43	0,82±0,25	N/A	0,04±0,04	1,41±0,80	0,12±0,05
Trem2	**RAW 264.7**	1,13±0,45	1,62±0,46	0,14±0,10	0,96±0,45	0,44±0,16	0,44±0,09
	**J774**	2,81±1,98	1,72±0,77	N/A	0,28±0,09	1,15±0,33	0,75±0,41
Mmp9	**RAW**	1,68±0,94	3,07±1,45	5,49±3,82	7,09±2,15**	4,28±1,45**	6,12±1,38**

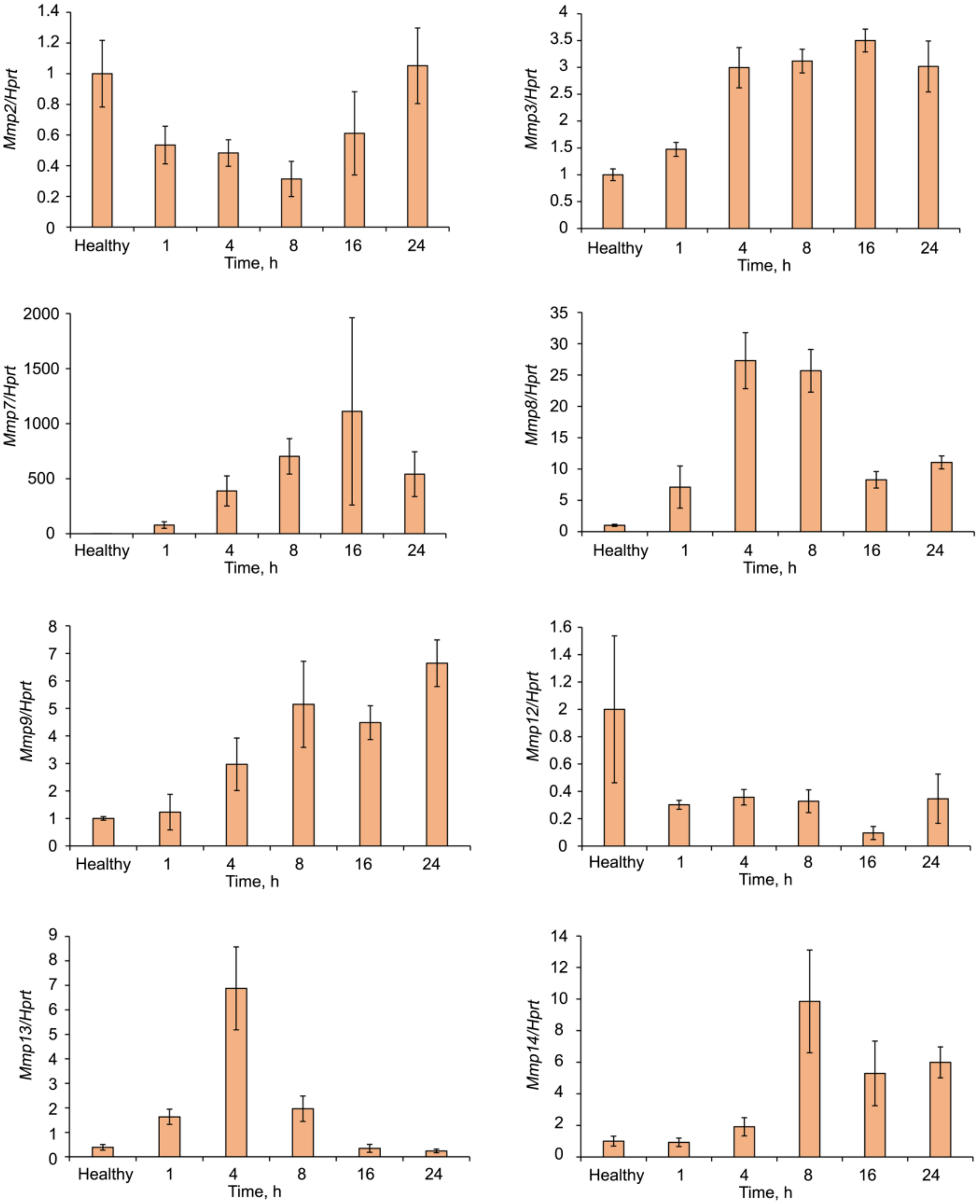

Figure S1. The expression levels of *Mmp2*, *Mmp3*, *Mmp7*, *Mmp8*, *Mmp9*, *Mmp12*, *Mmp13*, *Mmp14* in the lung tissue of LPS-challenged mice measured by RT-qPCR. Expression levels were normalized to the expression level of *Hprt* used as the reference gene. Three to five samples from each experimental group were analyzed in triplicate. The data are shown as mean ± standard deviation.

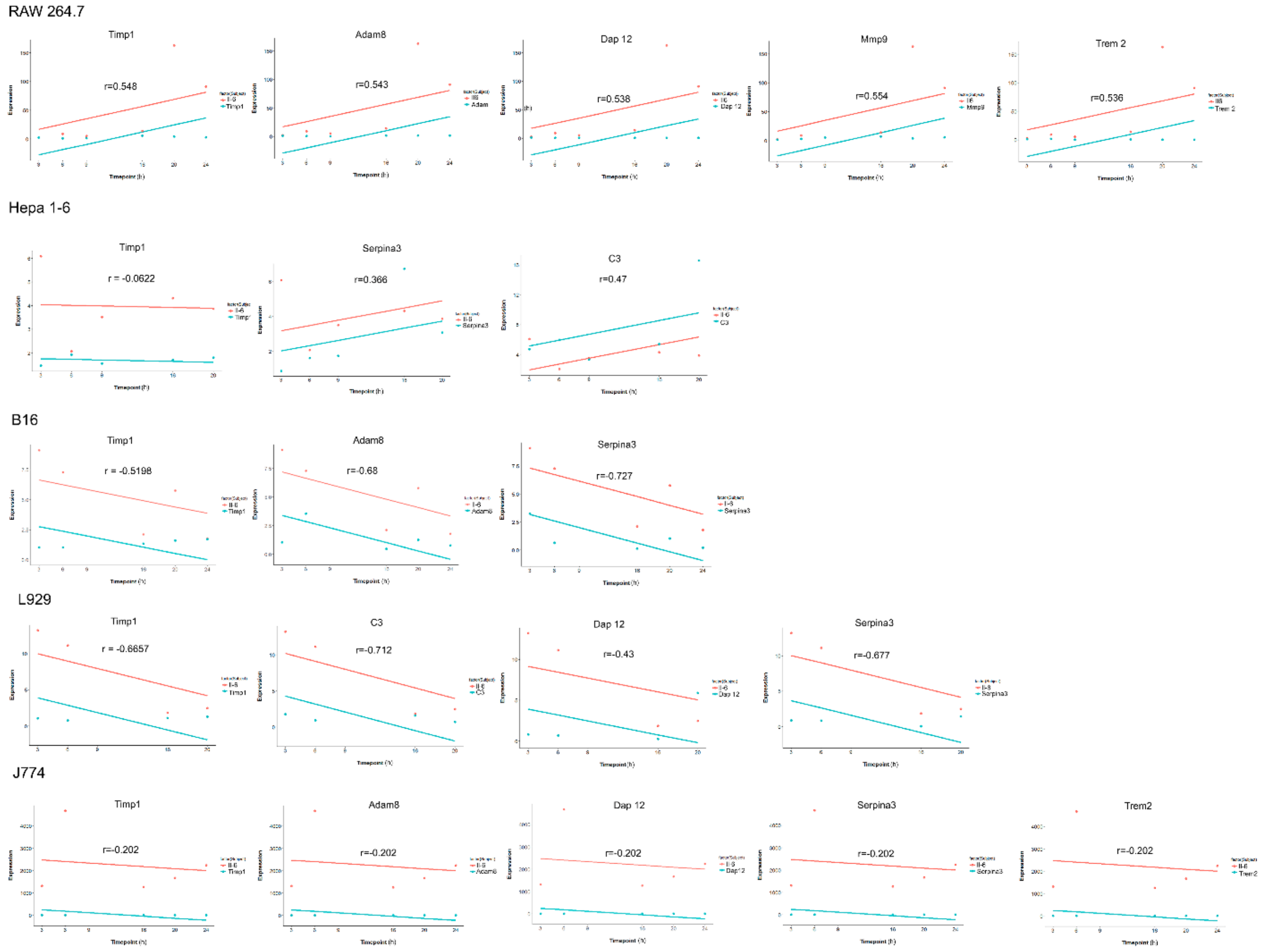

Figure S2. Correlation analysis of gene expression levels in different cell lines after LPS stimulation. Correlation coefficients were calculated using rmcorr package in R. Plots were constructed using the ggplot2 package in R. X and Y-axis breaks on the plots were intro-duced using the ggbreak package in R. Correlation coefficients were determined as follows: strong correlations $0.70 \leq r \leq 1.00$, moderate correlations $0.30 \leq r \leq 0.69$, weak correlations $0.01 \leq r \leq -0.29$.

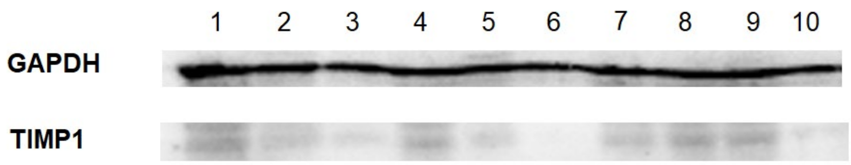

Figure S3. TIMP1 protein level in RAW 264.7 cells transfected with Lipofectamine 2000 4 days before measurement, 16 hours after LPS stimulation. Representative image of a Western blot: samples of the cells treated with siTIMP1_2m (lines 2, 3, 5, 6), siSCR (line 8), LF (line 9), LPS stimulated control (lines 1, 4, 7), and non-treated, non-stimulated control (line 10).

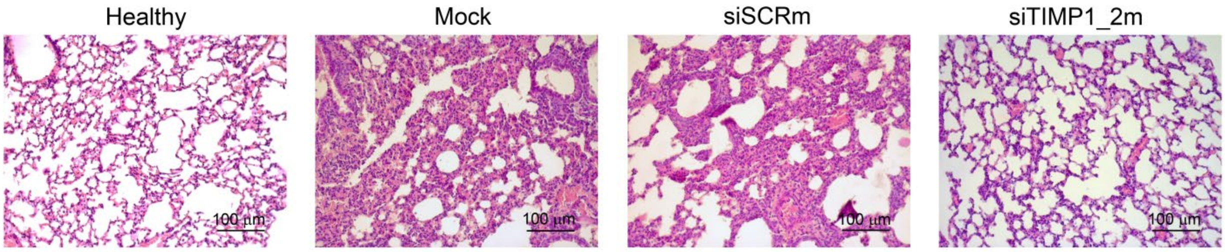

Figure S4. Representative histological images of lung tissue of healthy mice and LPS-challenged mice without treatment and after siTIMP1 administration outside the foci of severe inflammatory infiltration used for calculation of the volume densities of inter-alveolar septa reflection the interstitial edema. Haematoxylin and eosin staining. Original magnification ×200.
